# Anatomical Features and Material Properties of Human Surrogate Head Models Affect Spatial and Temporal Brain Motion under Blunt Impact

**DOI:** 10.3390/bioengineering11070650

**Published:** 2024-06-25

**Authors:** Michael Hanna, Abdus Ali, Prasad Bhatambarekar, Karan Modi, Changhee Lee, Barclay Morrison, Michael Klienberger, Bryan J. Pfister

**Affiliations:** 1 Center for Injury Biomechanics, Materials and Medicine, Department of Biomedical Engineering, New Jersey Institute of Technology, Newark, NJ 07102, USA; mh7376@nyu.edu (M.H.); asa58@njit.edu (A.A.); pvb7@njit.edu (P.B.); khm22@njit.edu (K.M.); 2 Neurotrauma and Repair Laboratory, Department of Biomedical Engineering, Columbia University, New York, NY 10027, USA; cl3426@columbia.edu (C.L.);; 3 The Army Research Laboratory, Aberdeen Proving Grounds, Aberdeen, MD 21005, USA; michael.kleinberger.civ@mail.mil

**Keywords:** human head surrogate, TBI, brain motion, injury thresholds

## Abstract

Traumatic brain injury (TBI) is a biomechanical problem where the initiating event is dynamic loading (blunt, inertial, blast) to the head. To understand the relationship between the mechanical parameters of the injury and the deformation patterns in the brain, we have previously developed a surrogate head (SH) model capable of measuring spatial and temporal deformation in a surrogate brain under blunt impact. The objective of this work was to examine how material properties and anatomical features affect the motion of the brain and the development of injurious deformations. The SH head model was modified to study six variables independently under blunt impact: surrogate brain stiffness, surrogate skull stiffness, inclusion of cerebrospinal fluid (CSF), head/skull size, inclusion of vasculature, and neck stiffness. Each experimental SH was either crown or frontally impacted at 1.3 m/s (3 mph) using a drop tower system. Surrogate brain material, the Hybrid III neck stiffness, and skull stiffness were measured and compared to published properties. Results show that the most significant variables affecting changes in brain deformation are skull stiffness, inclusion of CSF and surrogate brain stiffness. Interestingly, neck stiffness and SH size significantly affected the strain rate only suggesting these parameters are less important in blunt trauma. While the inclusion of vasculature locally created strain concentrations at the interface of the artery and brain, overall deformation was reduced.

## 1. Introduction

Sixty nine million individuals worldwide are estimated to suffer from traumatic brain injury (TBI) [1]. In the United States, TBI affects more than 2.8 million patients per year, and the rate of TBI emergency visits increased nearly by 54% between 2006 and 2014 [2]. TBI is the most prevalent cause of death in adults under 45 years and the highest cause of long-term disability [3,4,5,6]. Around USD 48.3 billion is spent in the US each year on TBI [7,8,9]. Of all TBI, 78% are caused by blunt injury to the head including unintentional falls, struck by or against an object, and motor vehicle crashes [2]. This includes contact sports and the military with high risk of TBI where 6% of college football players and an estimated 15% of deployed US military personnel have had a concussion or mild TBI [10,11,12]. 

For closed head injuries, it is believed that TBI is a result of mechanical loading to the head that leads to damaging motions throughout the brain [4,13]. The resulting rapid deformation of the brain tissue directly induces injury to neurons and their axons [14,15,16,17,18,19,20,21,22]. The biomechanical parameters of any blunt TBI vary significantly with regard to speed of impact, magnitude of force, and duration of the impact, as well as the direction and location of the impact on the head [23,24,25]. Therefore, the biomechanical nature of the impact will result in different brain deformations and likewise the severity of injury to the brain [26].

Experimental and computational approaches have been used to study how blunt impacts lead to brain motion in order to assess the risks of TBI. While brain motions cannot be measured within human subjects, current approaches must translate head kinematic measurements to the resulting motion of the brain using matched computational models. For instance, wearable devices have been designed to measure the real-time rigid body kinematics of the head on human subjects [27,28]. To predict the response of the brain tissues, measured head kinematics are used in finite element simulations [29,30,31]. To link brain motion to injury, the computed distribution of stresses and strains are related to estimates based on experimental animal and cadaveric data, mechanical testing of tissue samples, and reconstructions of real-life injury scenarios [32]. Indeed, finite element models have the capability to modify anatomical variables as well as material properties to evaluate their effects on the model outcome [4]. While finite element models provide excellent spatial and temporal resolution with anatomic accuracy, they are indeed a predictive model and need to be validated against experimental data [33,34]. Since non-computational experimental questions cannot always be addressed in a human subject, surrogates of the human head have been used to provide experimental data [31,35,36]. 

To best understand how brain deformations lead to injury, there remains a need to measure the response of the brain to blunt impacts. In the laboratory, blunt impact studies on the human head have been performed using instrumented surrogate head (SH) models. Recent advancements include a surrogate head designed with accelerometers, strain gages, and pressure transducers to measure skull deformation, intracranial pressure, and head kinematics, respectively [27,37,38,39]. The biofidelity of SH models has been addressed with postmortem human subjects to measure skull deformation and fracture, but on a very limited basis [35,40,41]. These measurements, however, still require a computational model to estimate brain deformations. Only a few approaches have been used to directly measure intracranial motions using human scale SH models. In a full closed skull model, imaging intracranial motions at the high rates associated with TBI has only been achieved using high-speed X-ray imaging of neutral density markers to estimate average strains at discrete locations [35,42,43]. The use of neutral density targets within a large volume, however, limits the spatial resolution of the method. 

To overcome the limitations of imaging at high rates of speed and spatial resolution, direct visual approaches have been used. Postmortem human subjects’ skulls, cut in half, have been filled with synthetic gels and a high-speed camera used to film the motions of a marker grid on the gel [44,45]. These early studies were used to predict the shear strain associated with inertial, rotational head injury which are still used as injury thresholds today [32]. Collectively, however, SH models have not been used to study the relationship between the mechanical parameters of blunt head impacts and the spatial and temporal deformation patterns in the brain.

To address the gap in available experimental methods to measure intracranial motions under blunt impact, we previously developed a method to measure the spatial and temporal strains within the sagittal plane of a half SH under blunt impact [26]. This model has the advantages of quickly adapting and testing variations in anatomical features and material properties as well as variations in blunt impact conditions. The objective of this study was to examine material properties and anatomical features of an SH model including surrogate brain stiffness, surrogate skull stiffness, inclusion of cerebrospinal fluid (CSF), head/skull size, inclusion of vasculature, and neck stiffness. Each feature was varied and the spatial and temporal variations in brain motion were analyzed under blunt impact conditions. The results reveal the relative importance of each parameter to experimental TBI modeling.

## 2. Methods

**Figure 1:**
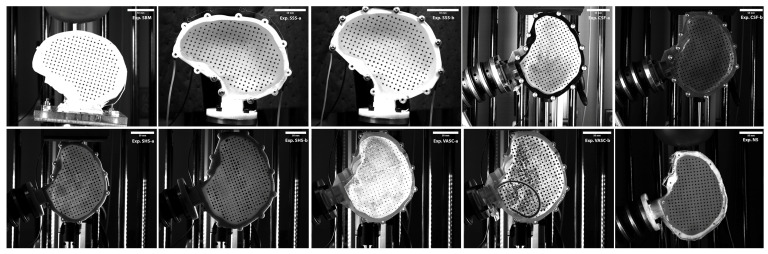
Images of the experimental setup. Exp.SBM shows the PVC skull in crown impact used to study the effect of the surrogate brain material. Exp.SSS-a shows the unstiffened ABS skull in crown impact used to study the effect of surrogate skull stiffness. Exp.SSS-b shows the stiffened ABS skull in crown impact used to study the effect of surrogate skull stiffness. Exp.CSF-a shows the VeroClear skull in frontal impact with CSF used to study the effect of CSF. Exp.CSF-b shows the VeroClear skull in frontal impact without CSF used to study the effect of CSF. Exp.SHS-a shows the 10th percentile ABS skull in frontal impact used to study the effect of SH size. Exp.SHS-b shows the 90th percentile ABS skull in frontal impact used to study the effect of SH size. Exp.VASC-a shows the VeroClear skull in frontal impact without vasculature used to study the effect of vasculature. Exp.VASC-b shows the VeroClear skull in frontal impact with vasculature used to study the effect of vasculature. Exp.VASC-b shows the VeroClear skull in frontal impact with vasculature used to study the effect of vasculature. Exp.NS shows the ABS skull in frontal impact to study the effect of neck stiffness.

Surrogate heads (SH) were designed and constructed to test the effects of six anatomical and material property variables. Table 1 lists the details of each blunt impact scenario where the experiment acronym will be used throughout the text to refer to the experiment. Figure 1 illustrates each SH and the blunt impact configuration. Supplemental Figure S4 provides a flow-chart detailing the design process to develop each SH and experiment that are explained below.

### 2.1. Characterizing Brain Surrogate Stiffness (Exp. SBM)

Many groups have used synthetic gels to simulate brain tissue [40,46,47,48,49,50,51,52], with reported tensile moduli between 15–125 kPa varying with composition and manufacturer [52,53,54]. For this study, a 5%, 10%, and 20% synthetic ballistics gel (Clear Ballistics) was used to experimentally assess the effects of stiffness on brain motion under blunt impact (Surrogate Brain Material experiment, SBM), Table 1. These synthetic gels are composed of polystyrene, polyisoprene, and mineral oil (formulations are proprietary). As previously described, the ballistic gel was set into a half skull to the midsagittal line and a grid of uniform black markers 7 mm apart was placed onto the gel surface for high-speed visualization of motion under blunt impact [26].

To characterize the Clear Ballistic gel viscoelastic properties, we used an indentation test developed for the testing of brain tissue [55,56]. In total, 5%, 10%, and 20% gel samples were formed onto 35 mm diameter and 3 mm thick cylinders. The indentation protocol involved 10 s of the initial hold and then 40 μm indentation + a 20 s hold while the force was measured (stress relaxation under constant strain). Tests were carried out for three different regions of each sample using a cylindrical indenter. For the analysis, stress relaxation was modeled as a 3-term Prony series; the load was predicted from the hereditary integral fit to the experimental load using constrained least-squares optimization. 

### 2.2. Changing the Surrogate Skull Stiffness (Exp. SSS)

**Figure 2:**
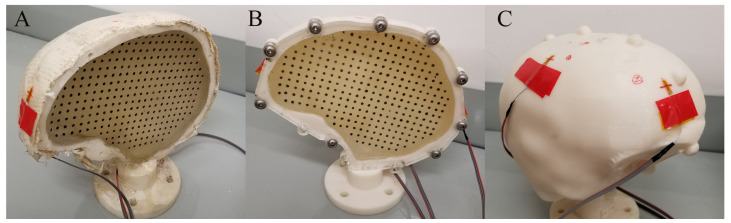
(**A**) Unstiffened half surrogate skull (**B**) Stiffened surrogate skull (**C**) Full surrogate skull.

The half skull design was created to allow for visualization of markers. However, it creates a limitation of our model as the bending stiffness of the skull at the point of a crown impact is reduced compared to a full skull, Figure 2. To design the proper bending stiffness, the skull deformation under crown impact was tested with a full surrogate skull filled with the same 20% ballistics gel. Results were used to develop a half skull model with a similar deformation response. Three different surrogate skulls were 3D printed with ABS material (Figure 2): (1) half skull unstiffened, (2) half skull stiffened with a bolted 0.25″ (6 mm) acrylic window, and (3) last a full surrogate head. For the stiffened half skull, we adopted a 3D skull model based on a CT scan and used CAD software (version 2.0.19215 x86_64) to add mounting holes to the 3D skull model. Then, using a Stratasys F370 3D printer, we printed the modified skull models out of ABS (exp. NF, SHS, and SSS), and the acrylic window was laser cut to match the hole patterns on the skull. All surrogate skulls were 3D printed from the same material, on the same 3D printer, and with the same 3D printing parameters.

Uniaxial strain gages (Omega KFH-6-350-C1-11L3M3R) were placed on three locations of the SH (front, crown, and rear), Figure 2. The upper side of strain gauge has a marking which was used for alignment. Strain gauges were mounted 10 mm from the cutting plane and at the fixed distance from the coronal plane. Data recording was carried out using National Instrument’s Compact Data Acquisition Chassis (NI cDAQ-9188) along with NI-9237. The strain gage measurements were initially validated against a simple cantilever beam, and the measured readings matched the calculated values within 2% of analytical solutions from measured deflections. Data were acquired at a sampling frequency of 50 kHz in LabView, and data were analyzed using MATLAB. 

### 2.3. Creating a CSF Layer (Exp. CSF)

In our previous work, the surrogate brain was cast directly inside the surrogate skull creating a no-slip boundary between the gel and inner skull surface. While the interface between the brain and skull is complex, the boundary can slip [57,58,59]. To assess the effects of a slipping boundary on the deformation of the brain under blunt impact, the SH was designed to include a CSF layer between the brain and skull. 

A separate surrogate brain was molded in a 98% scaled down 3D printed skull cavity, removed, and placed inside a full-size surrogate skull leaving a 2 mm space between the brain and inner surface. To create a watertight seal, a 0.25″ (6 mm) acrylic plate was laser cut to match the hole patterns on the skull, and a rubber gasket was laser cut and glued onto the acrylic plate to ensure water tightness. A port was created in the surrogate skull to inject water, and the volume of injected water was recorded. Water has been used as a simulant for CSF as its viscosity differs little from water [57,59]. The surrogate brain molds were scaled until approximately 70 mL filled the space (50% of the CSF volume), and the distance between the skull and the molded ballistic gel brain was approximately 2 mm [57,59,60]. Since 3D printed skulls of ABS were not watertight, we switched SH material to VeroClear printed on an Objet Prime V3 (Stratasys).

### 2.4. Creating Different Surrogate Head Sizes (Exp. SHS)

CT scan based skull models were scaled using CAD software, and measured head breadth was compared with published anthropometric data of US civilians [61]. Three SH sizes were 3D printed in 10th, 50th, and 90th percentile male head sizes. The SH size used in each experiment is summarized in Table 1. 

### 2.5. Creating Vasculature in SH (Exp. VASC)

The anterior cerebral artery was selected because it is a major artery in the brain, supplying blood to the frontal, parietal and cingulate cortex, and corpus callosum [62]. To simulate the anterior cerebral artery in experiment VASC, a rubber silicone tube 4.8 mm (3/16″) in diameter with a durometer hardness of 35 A was used. The 35 A shore hardness rubber silicone has an estimated tensile modulus of 1.39 MPa according to the A. N. Gent equation [63,64,65]. This calculated tensile modulus falls within range of reported artery stiffness [66]. 

### 2.6. Altering the Surrogate Neck Stiffness (Exp. NS)

**Figure 3:**
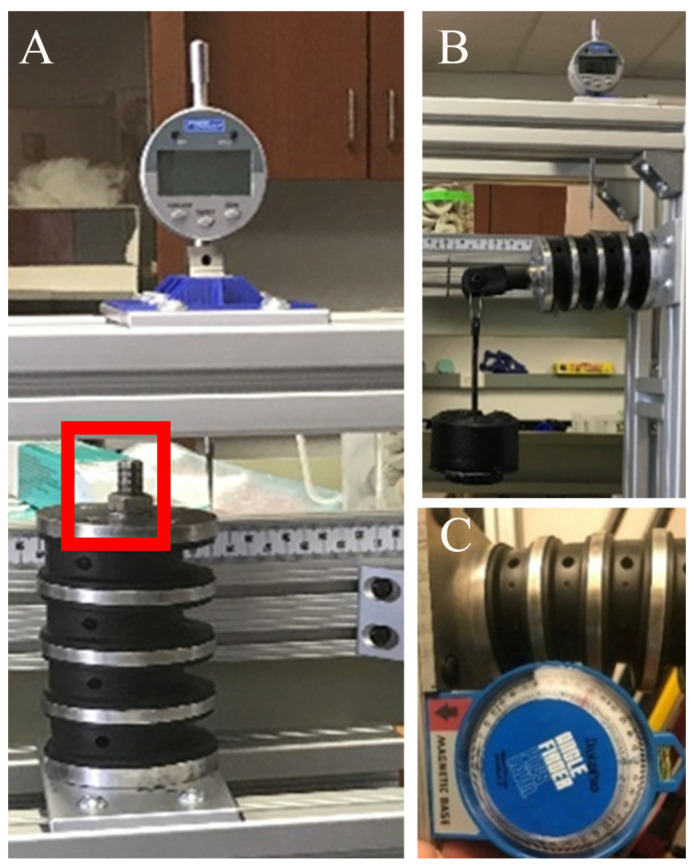
Hybrid III neck calibration. (**A**) Dial indicator being used to measure Hybrid III neck compression. (**B**) weights being mounted on the Hybrid III neck to measure stiffness. (**C**) Hybrid III neck deformation is being measured using a protractor.

The Hybrid III neck is made of butyl rubber with steel end plates and has a steel cable that runs through its center [67]. Changes in stiffness are achieved by tightening the cable and compressing the Hybrid III neck, Figure 3. Tightening the nut changes the neck’s overall height. Using a dial indicator, the change from the nominal height of the Hybrid III neck was set to three heights (three levels of neck stiffness)—1.27 mm (0.050″), 3.81 mm (0.150″), and 6.35 mm (0.250″). To calibrate neck stiffness, the neck was mounted in a cantilever position, and weights were added to the extremity. Neck deformation was measured using a protractor, and neck stiffness was calculated in Nm/° using Equation (1), where w is the mass of the weight in kg; d is the moment arm and θ in the neck deformation in degrees [68].

$$neck\;stiffness=\frac{wgd}{\theta }$$

Equation (1) neck stiffness formula in Nm/°.

To compare our data to published studies of active neck stiffness [69], we also calculated the neck stiffness in N/° using Equation (2), where w is the mass of the weight in kg and θ in the neck deformation in degrees.

$$neck\;stiffness=\frac{wg}{\theta }$$

Equation (2) neck stiffness formula in N/°.

### 2.7. Surrogate Skull Mechanical Properties

Materials used for the surrogate skulls varied based on the SH development and experimental design. For this study, PVC skulls (Anatomy Warehouse) were used in the original SH design [26]. ABS was used for skulls that were 3D printed, and VeroClear material was used for water tight 3D printed skulls. For PVC (exp. SBM), the reported tensile modulus was between 2.5 and 5 GPa and a fracture strain of 0.030 [70,71]. For Stratasys VeroClear (exp. CSF and VASC), the reported tensile modulus was between 0.9 and 2.5 GPa, and the fracture elongation was from 0.0098–0.083 [72,73]. Lastly, for Stratasys ABS (exp. NF, SHS and SSS), the reported tensile modulus was 0.9–1.9 GPa, and the fracture elongation was from 0.017–0.035 [74,75]. As for the human cranial bone, the reported tensile modulus was between 0.45 and 10 GPa, and the fracture elongation was from 0.030–0.15 [76,77,78,79,80]. These data suggest that PVC, VeroClear, and ABS fall within measured properties of the human cranial bone. 

### 2.8. Head Impact Testing Parameters

The SH models were mounted on the drop tower (Cadex Inc., Saint-Jean-sur-Richelieu, QC, Canada) in a “front” and “crown” impact orientation. The impact speed for all experiments was 1.34 m/s (3 mph) with either a head-form or flat impactor. The EN960 Magnesium head-form (assembled weight of 4.54 kg) is an impactor that is supplied with the drop tower system (Cadex). While the head-form is representative of an impact to a head, using it as an impactor to our surrogate head-form creates a head-to-head impact configuration. This setup is not representative of other head impacts such as a fall on a flat surface. In addition, we found that it caused frequent surrogate skull fractures especially in the VeroClear SH likely due to the impact forces concentrated to the point of impact. To represent a blunt impact onto a flat surface, we custom machined an aluminum flat impactor that weighed 1.75 kg and glued a 6 mm (0.25″) flat rubber padding to it. The details of each experiment are presented in Table 1.

### 2.9. Motion Tracking and Strain Calculations

All drop tower experiments were recorded with a high-speed camera (UX100 M3; Photron, San Diego, CA, USA) at 2000 fps and repeated 4 times. Motion tracking and strain calculations were carried out as previously published [26]. In brief, the motion of the marker grid was tracked from videos generating *x* and *y* coordinates at every video frame (ProAnalyst, Xcitex Inc., Woburn, MA USA). A custom MATLAB script was used to transform the marker displacements into a finite deformation tensor of the midsagittal plane of the surrogate brain and calculate the Lagrangian finite strain tensor E, which mapped the strain field spatially and temporally for each video frame. Transformation equations were used to convert E to principal strains $\varepsilon_{1}$, $\varepsilon_{2}$, and γmax. 

To compare motions and strains between experimental designs, the number of markers was kept the same on each surrogate head. This meant that the total number of segments in each SH was also the same. Strain concentration factor and strain rate fold increases were calculated by dividing the resultant principal shear strain in each segment. MATLAB was used to create heat maps of strain concentration or strain rate fold increase. 

### 2.10. Relating Surrogate Brain Deformation to Injury Risk

The literature has proposed that a head injury risk threshold of 15% shear stain correlated with injury in preclinical models [81,82]. Using this approach, the percentage area of surrogate brains with shear strains higher than 0.15 for each experiment was calculated [26]. If the experimental outcome had all areas with strains less than 0.15, we used a threshold of 0.075 and identified these areas of high strain. 

### 2.11. Statistical Analysis

To plot the heatmaps, the average maximum principal strain, strain impulse, and strain rate data of the three trials were calculated per marker grid segment. To compare heatmaps in terms of maximum principal strains, strain impulse, and strain rate, two different statistical approaches were used. First, an ANOVA (anova1 MATLAB function) was performed to reject the null hypothesis that all group means were equal. Second, the average maximum principal strain, strain impulse, and strain rate data per heatmap were calculated, and data were presented as means and standard deviations.

## 3. Results

### 3.1. The Effects of Surrogate Brain Stiffness on the Development of Strain

**Figure 4:**
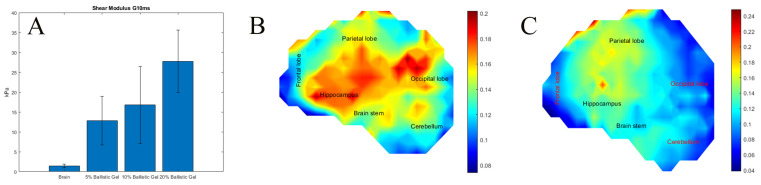
(**A**) Measured shear modulus of ballistics gel compared with human brain. (**B**) Maximum shear strain heat map of 5% ballistics gel surrogate head. (**C**) Maximum shear strain heat map of 20% ballistics gel surrogate head.

The shear moduli of the ballistics gel concentrations were measured and compared to moduli measured from brain tissue. Measured shear moduli (G(t)) were fitted to a three-term Prony series, and plots for G(t) are shown in the Supplementary Material, Supplemental Figures S1 and S2. Dynamic measurements of brain shear modulus remained constant during the first 10 ms of deformation, and therefore, this time period was used to compare to the ballistic gel stiffness, where for the 5% gel G_10ms_ = 12.85 kPa ± 6.1, the 10% gel G_10ms_ = 16.79 kPa ± 9.6 and the 20% gel G_10ms_ = 27.75 kPa ± 7.8. The 5% gel was stiffer than the brain tissue measured using the same method, G_10ms_ = 1.4 kPa ± 1.45 (Figure 4A) [55].

To test the degree to which surrogate brain stiffness affects the development of strain, we impacted the SH made with 5%, and 20% ballistic gel in the crown impact orientation. The 20% gel has been used by us and others in head models, while 5% is closer to the measured brain stiffness [40,50,51,52,54,83,84,85,86]. Maximum shear strain was 1.24 times higher in 5% SH 0.14261 ± 0.026 compared with 20% SH 0.1148 ± 0.033 (*p* < 0.01 by ANOVA F (466) = 102), Figure 4. The strain rate was 1.55 times higher in 5% SH 24.66 ± 17.59 s^−1^ compared with 20% SH 15.81 ± 8.98 s^−1^ (*p* < 0.01, F (466) = 46.18). Despite these differences, the strain impulse was not significantly different between the groups over the impact period, Figure S3. The average strain impulse was 2.29 ± 0.49 ms in the 5% SH compared with 2.27 ± 0.79 ms in the 20% SH. High risk areas (shear strain > 0.15) in the 5% SH were the parietal lobe, hippocampus, brain stem, and occipital lobe, constituting 45.18% of the total surrogate brain area, Figure 4. There were fewer high-risk areas in the 20% SH: parietal lobe and hippocampus constituting 15.41% of the total surrogate brain area.

### 3.2. The Effects of Skull Stiffness on the Spatial Development of Strain (Exp. SSS) 

**Figure 5:**
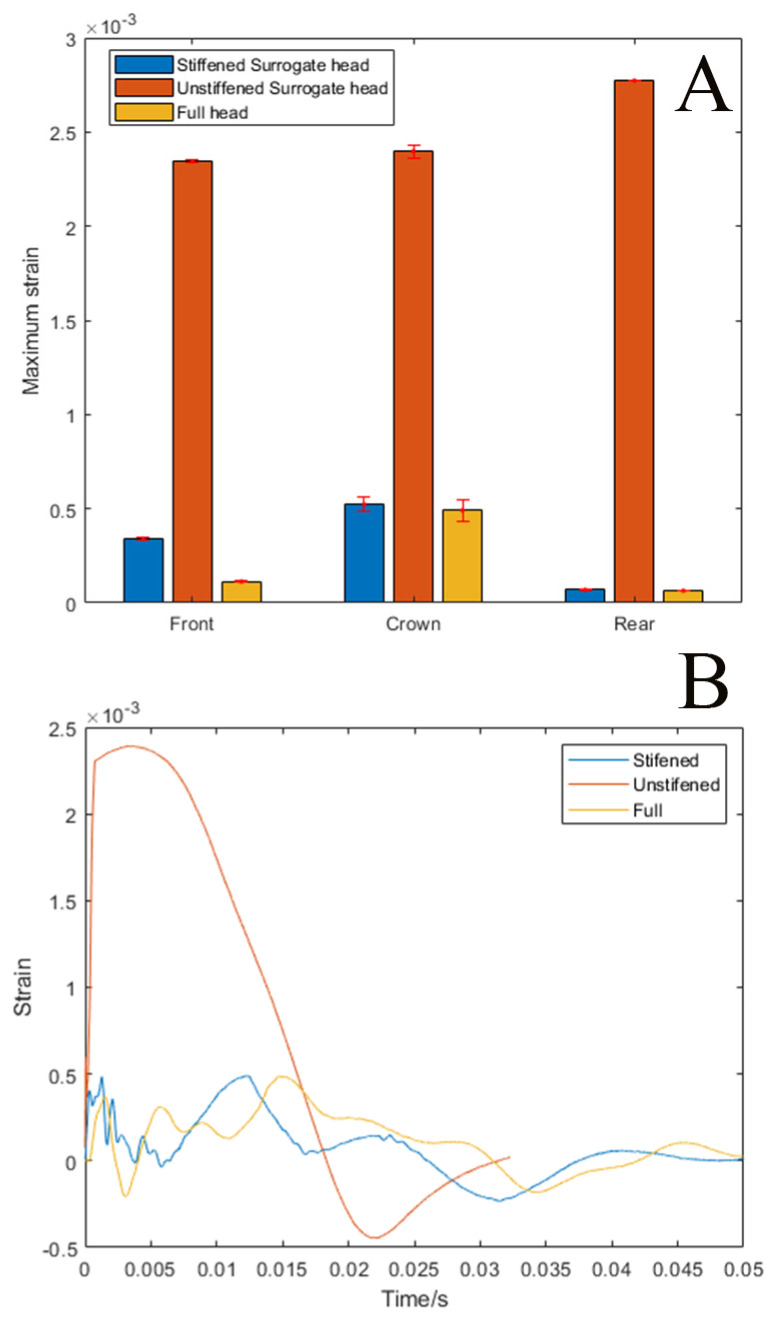
(**A**) Bar graph of maximum strain gage measurements in the front, crown, and rear positions for stiffened, unstiffened, and full surrogate head. (**B**) Strain time course of the crown strain gage for stiffened, unstiffened, and full surrogate head.

In our previous work, our observations of brain deformation under crown impact suggested it was largely due to skull flexion. To test this, we compared three surrogate skull models, the half surrogate skull, a full skull, and a half skull stiffened with an acrylic window to replicate the full skull deformation. Skull deformation from a crown impact was measured with strain gauges placed on the front and oriented along the sagittal axis and crown, oriented along the coronal axis, and rear oriented along the sagittal axis of the surrogate skull, Figure 2. Confirming our concern, the unstiffened half skull had significantly higher strains (5–15 fold) than the full surrogate skull. Our final stiffened half skull design was within the range of the full skull, Figure 5. For the rear strain gage, there was no significant difference between the full surrogate skull and the stiffened surrogate skull. For the crown position, the strain gage measurements were close, 6.41% higher in the stiffened skull (F (5) = 9.85, *p* < 0.05). While the front strain gauge measurement was 300% higher in the stiffened skull compared with the full skull (F (5) = 841.34, *p* < 0.001), it was much closer to the actual strain than the unstiffened skull. 

Surrogate brain deformation was compared between the two SH half skull models using the 20% ballistics gel. Maximum shear strains were 1.9 times higher in the unstiffened SH skull, 0.1368 ± 0.035, compared with the stiffened SH skull, 0.0714 ± 0.015 (F (505) = 735.97, *p* < 0.001). The strain rate was 1.37 times higher in the unstiffened SH, 15.30 ± 7.66 s^−1^, compared with the stiffened SH, 11.83 ± 11.17 s^−1^ (F (505) = 16.5, *p* < 0.001). The strain impulse was 2.3 times higher in the unstiffened SH, 2.03 ± 0.67 ms, compared with the stiffened SH, 0.87 ± 0.18 ms (F (505) = 707.47, *p* < 0.001). 

**Figure 6:**
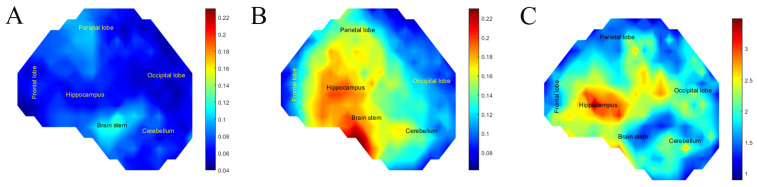
(**A**) Heat map of maximum shear strain for stiffened surrogate skull head (**B**) Heatmap of Maximum Shear Strain for unstiffened surrogate skull head (**C**) Heat map of shear strain fold increase between unstiffened and stiffened surrogate skull head.

To illustrate the spatial differences, we plotted a heat map of the maximum shear strain in each SH and calculated a strain fold increase between the stiffened and unstiffened SH. Areas with a fold increase higher than 2 were mainly in the hippocampus, Figure 6. Concerning high-risk areas (shear strain > 0.15) in the stiffened SH, they did not exist because all surrogate brain areas had shear strains lower than 0.15. However, the parietal lobe and brain stem had strains > 0.075, constituting 33.6% of the total SH area. While in the unstiffened SH, the high-risk areas were the parietal lobe, hippocampus, and brain stem consisting of 39.6% of the surrogate brain area. 

### 3.3. Effects of the CSF Layer on Brain Motion (Exp. CSF)

In our original design, the brain surrogate gel is formed in the skull creating a tight, no-slip boundary. Here, we considered the effects of a liquid boundary between the interior skull and the brain surrogate creating a slipping boundary. Here, the frontal impact orientation was chosen as it leads to the most rotational motion. The flat impactor was used as it led to more consistent impacts in the frontal orientation between SH models, and strains were calculated over the mid-sagittal plane. With the addition of a CSF layer, maximum shear strain was on average 2.7 times lower compared to the no-slip boundary (*p* < 0.01, F (522) = 1340.46). The SH with a CSF layer had an average maximum shear strain of 0.0617 ± 0.016 compared with 0.166 ± 0.044 in the SH without CSF. The average strain rate was, on average, 6.8 times lower in SH with CSF 8.46 ± 5.6 s^−1^ compared with SH without CSF 57.8±37.4 s-1 (*p* < 0.01, F (522) = 205). The average strain impulse, on the other hand, was, on average, 2.3 times lower, 0.656 ± 0.2 ms, in SH with CSF 1.49 ± 0.32 ms compared with SH without CSF (*p* < 0.01, F (522) = 1347.7). 

**Figure 7:**
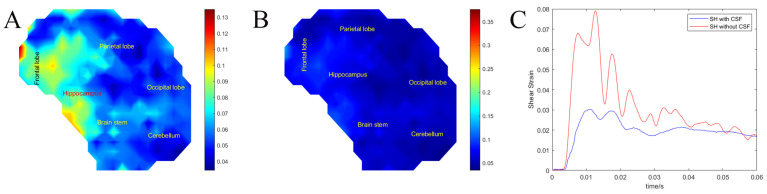
(**A**) Maximum shear strain heat map of SH without CSF. (**B**) Maximum shear strain heat map of SH with CSF. (**C**) Average surrogate brain shear strain over time of SH with CSF versus SH without CSF.

To illustrate the deformation of both models, we plotted shear strain heat maps on the midsagittal plane. Figure 7C shows the temporal response of each model showing that the CSF layer greatly attenuates the strains that develop in the brain under impact. Under the same impact conditions, high-risk areas (shear strain > 0.15) in the SH with CSF did not develop as shear strains were lower than 0.15. However, the frontal lobe, parietal lobe, and hippocampus had strains > 0.075, constituting 20.5% of the total SH area. The SH without CSF had high risk areas in the parietal lobe, hippocampus, and occipital lobe constituting 63.21% of the total SH area.

### 3.4. Surrogate Head Size (Exp. SHS)

Most TBI studies use the 50th percentile male skull size which does not represent the entire TBI population [87,88,89,90,91]. To assess the importance of individualization of skull size in our models, we created SH scaled to the 10 and 90th percentile male skull and measured brain motions from frontal blunt impacts. The maximum shear strain and the strain impulse were not significantly different between the two groups. The 10th percentile SH had an average maximum shear strain of 0.130 ± 0.035 compared with 0.125 ± 0.030 in the 90th percentile SH. The average strain impulse was 1.95 ± 0.43 ms in the 10th percentile SH compared with 1.93 ± 0.43 ms in the 90th percentile SH. Only the average maximum strain rate was 1.25 higher in the 10th percentile SH, 27.30 ± 18.77 s^−1^, compared with the 90th percentile SH, 21.90 ± 14.83 s^−1^ (F (663) = 17.05, *p* < 0.001). 

**Figure 8:**
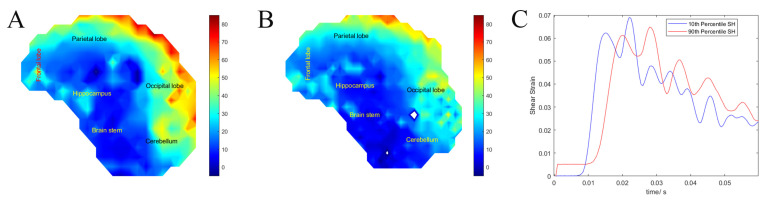
(**A**) Heat map of the shear strain rate for the 10th percentile surrogate head. (**B**) Heat map of the shear strain rate for the 90th percentile surrogate head. (**C**) Average surrogate brain shear strain over time of the 10th and 90th percentile surrogate head.

To illustrate the difference between the 10th and 90th percentile SH, we calculated strain rate distribution quantiles (25%, 50%, and 75%, respectively). For the 10th percentile SH, the strain rate quantiles were 12.86, 22.12, and 38.36, while for the 90th percentile SH, the strain rate quantiles were 10.14, 19.63, and 30.44, respectively. Finally, to compare the effect of head size on the model outcome, we plotted the shear strain heat maps for the 10th and 90th percentile SH and the average brain strain over time of injury, which clearly shows that only the strain rate was significantly different between the two different head sizes, Figure 8.

### 3.5. Vasculature (Exp. VASC) 

**Figure 9:**
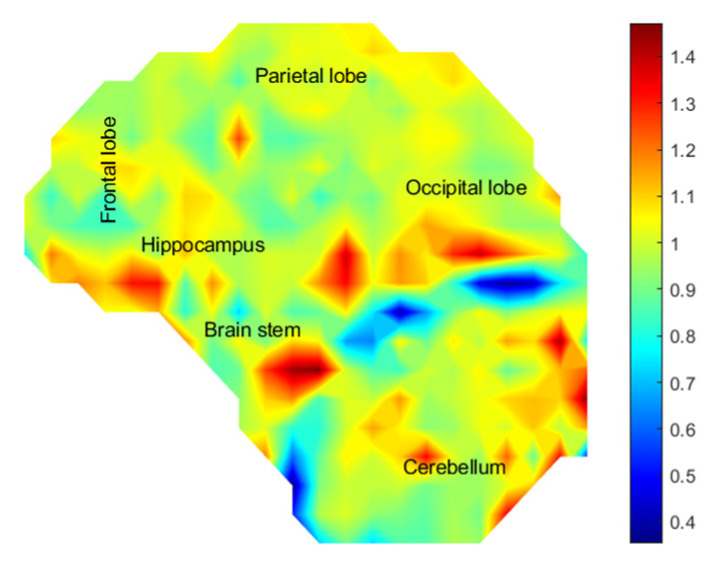
Heat map of the strain concentration factor of the surrogate head with vasculature compared with the surrogate head without vasculature.

Many finite element models have reported on the relative significance of the cerebrovascular network providing mechanical support to the brain [4,92,93,94,95,96]. Here, the presence of the major anterior cerebral artery on brain deformation was investigated. Interestingly, the presence of the anterior cerebral artery did not lead to significantly different deformation with the brain regions. The SH without vasculature had an average maximum shear strain of 0.1553 ± 0.0366 compared with 0.1526 ± 0.0422 in the SH with vasculature. The average shear strain rate was 15.90 ± 11.32 s^−1^ in the SH without vasculature compared with 14.36 ± 10.05 s^−1^ in the SH with vasculature. Last, the average strain impulse was 1.39 ± 0.31 ms in the SH without vasculature compared with 1.37 ± 0.37 ms in the SH with vasculature. While the presence of the artery did not change the overall motion of the brain, local concentrations of strains at the interface of the surrogate brain and artery are noticeable in the heat map, Figure 9. 

### 3.6. Neck Stiffness (Exp. NS)

The literature using finite element models and human subject testing has reported conflicting data on increased neck stiffness being protective from TBI [69,97,98,99,100,101]. We calibrated the hybrid III neck into three different levels of stiffness (low, medium, and high) and compared the injury outcome using the three different stiffnesses. The low stiffness neck had a stiffness of 1.4 ± 0.09 Nm/° or 7.17 ± 0.45 N/°. The medium stiffness neck had a stiffness of 1.7 ± 0.08 Nm/° or 8.73 ± 0.40 N/°. The high stiffness neck had a stiffness of 2.5 ± 0.15 Nm/° or 12.72 ± 0.78 N/°. The resultant stiffnesses were statistically significant (F (8) = 50.37, *p* < 0.001). 

**Figure 10:**
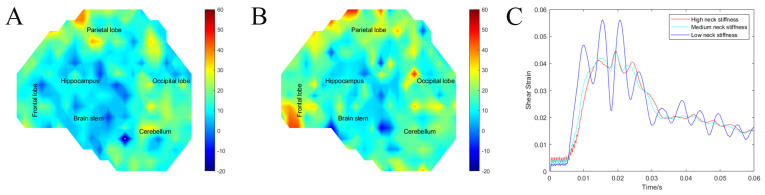
(**A**) Heat map of strain rate in high stiffness neck SH. (**B**) Heat map of strain rate in low stiffness neck SH. (**C**) Average surrogate brain shear strain over time of surrogate head with low, medium and high stiffness neck.

ABS 3D printed SH with 20% ballistics gel were tested under frontal impact to maximize rotation about the neck. The maximum shear strain and strain impulse were not significantly different between the three groups. The low stiffness neck SH had an average maximum shear strain of 0.0953 ± 0.017 compared with 0.0917 ± 0.016 in the medium stiffness neck SH and 0.0934 ± 0.016 in the high stiffness neck. For average shear impulse, the low stiffness neck SH had an average shear impulse of 1.012 ± 0.200 ms compared with 0.923 ± 0.156 ms in the medium stiffness neck SH and 0.952 ± 0.168 ms in the high stiffness neck. Only the strain rate showed a significant correlation with neck stiffness, the low stiffness neck SH had average maximum strain rate of 16.36 ± 9.99 s^−1^ compared with 14.87 ± 9.01 s^−1^ in the medium stiffness neck SH and 12.71 ± 8.42 s^−1^ in the high stiffness neck (F (793) = 20.7, *p* < 0.001), Figure 10. Finally, to compare the three groups of neck stiffness SH, we plotted the average surrogate brain strain over the time of injury, which clearly shows that only the strain rate was the significantly different parameter between the three groups.

## 4. Discussion

Surrogate head (SH) models offer an experimental approach to linking the external kinematics of trauma to damaging brain motions. Similar to computational models, the purpose of SH models is to evaluate the risk of a TBI based on the replication of the injury event. While SH models can be limited to the precise replication of anatomical features, boundary conditions, and material properties, they offer an experimental approach to study the effect of these parameters to intracranial motions and deformations [27,38,39]. This study considered the effects of anatomical variables (CSF, vasculature, and SH size) and material properties (brain surrogate material, skull stiffness, and neck stiffness) to surrogate brain motion from blunt impact. 

Here, we present a sensitivity study determining important factors to consider in biofidelic surrogate models for predicting brain deformation. Our findings suggest that the important experimental variables were the surrogate brain material, skull stiffness, and inclusion of a CSF layer. Of these three variables, the inclusion of CSF had the highest effect on surrogate brain deformation, followed by surrogate skull stiffness and surrogate brain material, Table 2. Interestingly, the surrogate brain material did not have a significant effect on strain impulse suggesting that the main contributor to the absorption of impact energy was the skull and the CSF layer. This observation can also be validated by comparing data from experiments SBM and SSS. In these experiments, two SH were impacted with the same speed and impactor geometry, differing only in surrogate skull material. In experiment SBM, the SH was made of PVC, which is stiffer than the ABS SH used for experiment SSS. Accordingly, the ABS SH had an average maximum shear strain of 0.1368 ± 0.0353, 1.19 times higher compared with 0.1148 ± 0.033 in the PVC SH, (F (491) = 50.52, *p* < 0.001). The average shear strain rate was not significantly different between both groups, 15.3031 ± 7.66 s^−1^ for ABS SH compared with 15.81 ± 8.98 s^−1^ for the PVC SH. Interestingly, the strain impulse in the PVC SH was 2.0306 ± 0.6676 ms, 11% higher than ABS 2.27 ± 0.79 ms, which might be attributed to the ABS greater toughness, (F (491) = 14.3, *p* < 0.001). High-risk areas (shear strain > 0.15) in the ABS SH were the parietal lobe, hippocampus, and brain stem consisting of 39.6% of the surrogate brain area. While in the PVC SH, high-risk areas were the parietal lobe and hippocampus, constituting 15.41% of the total surrogate brain area. The high-risk areas in both SH were the parietal lobe and hippocampus. The brain stem was also high risk in the ABS SH but not in the PVC SH.

Computational models have been used to predict brain areas of high risk to injury using the threshold of 15% shear strain linked to animal models of TBI [81,82]. The experiments in this study were grouped by impact orientation and found for crown impact; all experiments concluded that the parietal lobe is a high-risk area (shear strains > 0.15). In addition, 80% of the experiments had the hippocampus and brain stem as high-risk areas (shear strains > 0.15). As for frontal impact, 75% of the experiments had the occipital lobe as a high-risk area (shear strains > 0.15), and 50% of the experiments had the parietal lobe and hippocampus as high-risk areas (shear strains > 0.15). This indicates that impact orientation can be a major factor in injury outcome.

Anatomically, the boundary condition introduced by the CSF layer surrounding the brain allows for movement between the brain surface and the inner skull surface [31,36,102,103]. Our previous work with SH had a no-slip boundary due to molding the brain surrogate ballistic gel directly into the skull. To assess the importance of this boundary condition under blunt impact, this study considered the effects of a CSF layer creating an unrestricted slipping boundary. The realistic boundary is somewhere between these two conditions due to the connective tissue between the brain and skull and will additionally affect the motion of the brain from blunt impact. In this experiment, a 20% ballistic gel was used, which was 19.8 times stiffer than the human brain suggesting that strains in the real brain tissue are higher. The data for experiment SBM show that for a 2.15-fold decrease in surrogate brain stiffness, we only had a 24% increase in the average strain. 

The effects of vasculature, SH size, and neck stiffness were limited to an increase in strain rates and local strain concentrations. There was no significant effect on overall strain or strain impulse for the three parameters. Neck stiffness has gained attention due some speculation that females are more susceptible to TBI due to having weaker neck muscles [69]. Our strain data agree with other studies that used a finite element model to study the effect of neck stiffness on brain strains [99]. Importantly, SH with a stiffer neck had 28.7% lower strain rates than a less stiff neck, and the increase in strain rate was not isolated to a specific area. Accordingly, the TBI differences in men and women may not be related to neck stiffness. 

Our measurements of the Hybrid III neck stiffness were stiffer than the published data for passive neck stiffness (0.3–0.1 Nm/°)[68]. As for active stiffness, measured Hybrid III neck stiffness was on the upper range for published data (3.9–7.8 N/°). The stiffness was even further out of range for reported female neck stiffness [69]. These data indicate the need to lower stiffness surrogate necks to generate more realistic data in the future. 

There is concern over using the 50% male representation as a sole model for TBI [87,89]. Here, we found that the 10th percentile SH developed 24.6% higher strain rates than the 90th percentile SH. There was no significant difference in average shear strains between the two sizes. Locally, however, high-risk areas in the 10th percentile SH included the parietal lobe, occipital lobe, and cerebellum, constituting 36.23% of the total surrogate brain area. In the 90th percentile SH, the high-risk areas were the occipital lobe and cerebellum, constituting only 26.75% of the total surrogate brain area. While these results suggest that the size of an individual’s head does not make large differences in overall strain in the brain, studies have shown the importance of the strain rate to neuronal injury [104,105,106,107,108].

Many SH use homogenous materials to represent the bulk stiffness of the brain. In reality, the brain is a very heterogenous structure with grey/white matter, ventricles, connective tissue, and vasculature. This study considered the anterior cerebral artery to assess how large vasculature structures may affect brain motion. We found that average strain, strain rate, and strain impulse surrounding the surrogate artery were lower compared with the SH without vasculature; however, this drop was not significant. This finding agrees with finite element simulations that reported 13–46% reduction in brain strain [92,93,94,96]. The artery created a small, localized area of strain concentration. Further model development would be needed to investigate this concentration with a higher resolution. In addition, the high stiffness of the ballistics gel used in this experiment might be underestimating the influence of vasculature on the strain concentration in the brain; further studies are required to better understand the effect of vasculature. 

It is generally accepted that injury to the brain in TBI is initiated due to the stretching of nervous tissue. In vitro models have directly established the link between mechanical stretch and neuronal degeneration and death, dependent on both strain magnitude and strain rate [74,75,76,77,78,79]. Animal modeling has also supported these findings [17,109]. For studying this relationship between head kinematics of trauma and the resulting brain deformation in humans, the field has primarily relied on computational modeling. Here, we developed a human scale experimental platform to test the response of the brain to blunt impact with physical experiments. The SH created has the ability to alter anatomical and material properties with relative ease. Finally, we introduce the concept of strain impulse, representing the cumulative energy that has been absorbed by the brain. To our knowledge, the relationship between strain impulse and injury has not yet been examined in TBI models.

## 5. Conclusions

This study provides needed insight into anatomical and material property variables to consider in the design and experimentation of SH models under blunt impact. Inclusion of a CSF layer, which allows slip between the surrogate brain and the skull, had the highest impact on brain motion. Interestingly, the stiffness of the brain surrogate material had the smallest impact on brain motion. This study also concludes that skull flexion as a result of the skull stiffness is an essential parameter. Vasculature caused localized concentrations of strains at the interface of the surrogate brain and artery but no significant change in overall strain distribution. Neck stiffness and surrogate head size had little to no effects on intracranial motions. Overall, an SH should focus on replicating the correct skull stiffness and a biofidelic CSF interface between the brain and skull. While brain surrogate material properties are important, the data in this study suggest that the modulus can vary within the range of brain properties without a large effect on measured brain motions.

## Figures and Tables

**Table 1:** Impact orientation and shape of the impactor used for each experiment. Blunt impact speed was 1.34 m/s (3 mph) for all experiments.

Acronym	Experiment	SH Size Percentile Male	Surrogate Skull Material	Ballistic Gel Used	Impact Orientation	Impactor
SBM	Surrogate brain material properties	50th	PVC	5%, 10%, and 20%	Crown	Head-form
SSS	Surrogate skull stiffness	50th	ABS	20%	Crown	Head-form
CSF	CSF layer	50th	VeroClear	20%	Frontal	Flat
SHS	Surrogate head size	10th and 90th	ABS	20%	Frontal	Flat
VASC	Effect of Vasculature	50th	VeroClear	20%	Frontal	Flat
NS	Effect of neck stiffness	50th	ABS	20%	Frontal	Head-form

**Table 2:** Fold increase in injury parameters for experiments SBM, SSS, and CSF.

Injury Parameter	Surrogate Brain Material (SBM)	Surrogate Skull Stiffness (SSS)	Cerebrospinal Fluid (CSF)
Maximum shear strain	1.24	1.92	2.69
Shear strain rate/s^−1^	1.56	1.29	6.83
Shear strain impulse/ms	none	2.33	2.27

## Data Availability

The datasets generated and supporting the findings of this article are obtainable from the corresponding author upon reasonable request.

## Supplementary Materials

The following supporting information can be downloaded at: https://www.mdpi.com/article/10.3390/bioengineering11070650/s1, Figure S1: Fitted relaxation function parameters with 95% confidence intervals; Figure S2 G(t) for Human brain, 5% Gel, 10% Gel and 20% Gel, Figure S3 Average surrogate brain shear strain and strain impulse over time for 5% and 20% Gel surrogate head, Figure S4 flow-chart detailing the design process to develop each SH and experiment.
