# Supplementary material for: Anatomical Features and Material Properties of Human Surrogate Head Models Affect Spatial and Temporal Brain Motion under Blunt Impact
Source: Bioengineering (Basel). 2024 Jun 25;11(7):650. doi: 10.3390/bioengineering11070650 (PMC11273380; doi:10.3390/bioengineering11070650)
Supplement: Supplementary file 1 [file bioengineering-11-00650-s001.zip › bioengineering-3070380-supplementary.pdf]

Material	G_∞ (Pa)	G_1 (Pa)	τ_1 (s)	G_2 (Pa)	τ_2 (s)	G_3 (Pa)	τ_3 (s)
Human brain	223 ± 28	314 ± 32	5.75 ± 1.82	338 ± 44	0.287 ± 0.105	947 ± 103	0.0184 ± 0.0034
5% Gel	10200 ± 2500	1500 ± 317	13.8 ± 5.9	835 ± 89	0.727 ± 0.383	383 ± 130	0.0624 ± 0.0268
10% Gel	13700 ± 3800	1980 ± 680	19.2 ± 11.6	867 ± 317	1.10 ± 0.29	300 ± 0	0.0536 ± 0.0304
20% Gel	22600 ± 3300	2990 ± 358	15.1 ± 6.6	1920 ± 260	0.898 ± 0.291	300 ± 0	0.0800 ± 0

Figure S1 Fitted relaxation function parameters with 95% confidence intervals

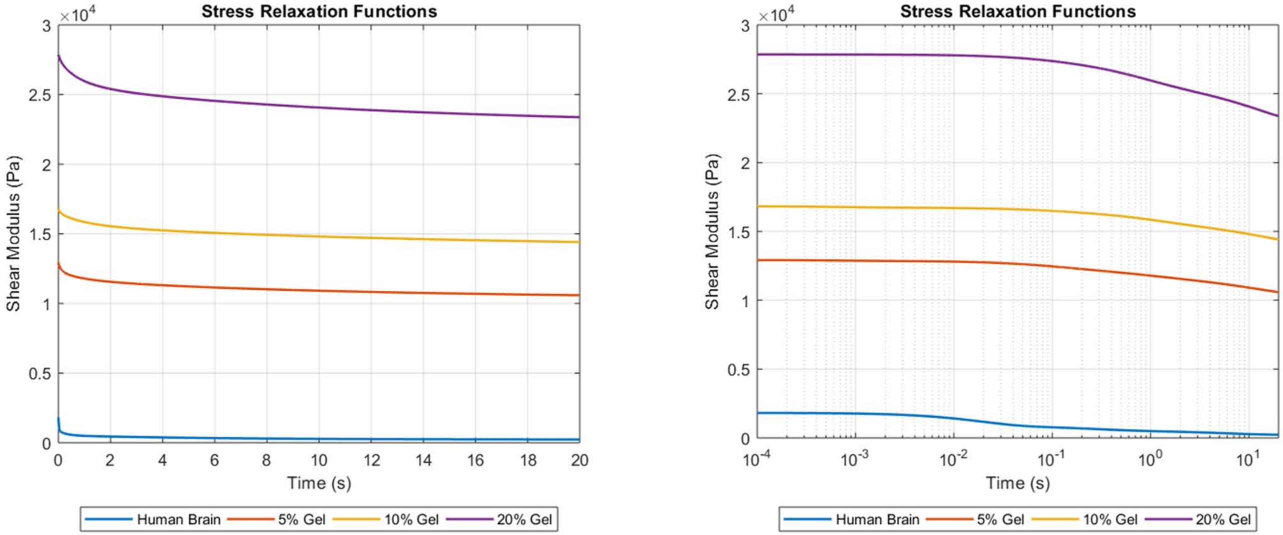

Figure S2 G(t) for Human brain, 5% Gel, 10% Gel and 20% Gel

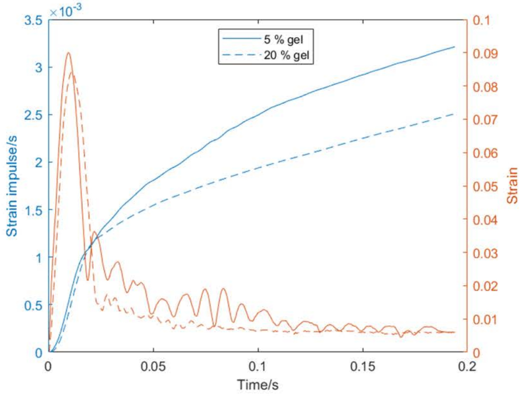

Figure S3 Average surrogate brain shear strain and strain impulse over time for 5% and 20% Gel surrogate head.

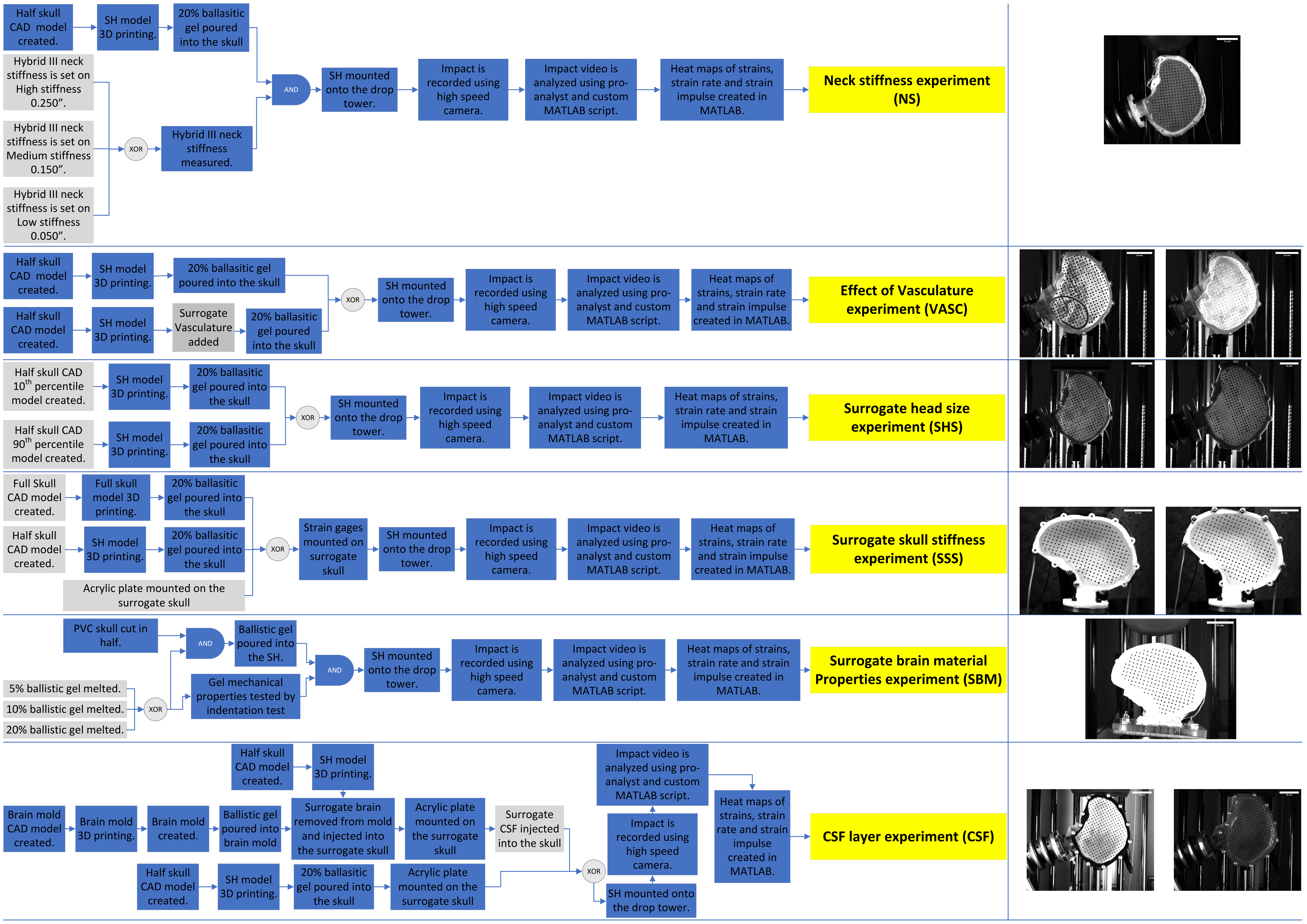

Figure S4 flow-chart detailing the design process to develop each SH and experiment
